# Life After Esports: A Grand Field Challenge

**DOI:** 10.3389/fpsyg.2020.00883

**Published:** 2020-05-05

**Authors:** Tim D. Smithies, Adam J. Toth, Eoin Conroy, Niall Ramsbottom, Magdalena Kowal, Mark J. Campbell

**Affiliations:** ^1^Department of Physical Education & Sport Science, University of Limerick, Limerick, Ireland; ^2^Lero, The SFI Centre for Software Research, University of Limerick, Limerick, Ireland

**Keywords:** video-games, career transitions, esport retirement, cognitive abilities, digital abilities

## Introduction

Esports has experienced unprecedented growth recently and with it, the proposition for aspiring gamers to pursue a professional esport career has become increasingly attractive. “Esports” are video-games played competitively (and often professionally) through the means of cyberspace (Campbell et al., 2018) and are an important fixture in the overall gaming industry, which is estimated to be worth more than 120 billion US$ (Takahashi, 2020). The exponential rise in popularity has led to the inclusion of two esports (Rocket League and Street Fighter V) in an International Olympics Council sanctioned tournament before the 2020 Tokyo Olympic games (Martinello, 2019).

Despite the appeal of esports as a profession, aspiring esport athletes face many obstacles that can threaten their prospective career timespan, and present post-career difficulties. To date, very limited formal exploration exists into this challenge; thus, this grand field challenge aims to explore the difficulties faced by esport athletes. It also highlights the unique skillsets and experience acquired during a professional esports career, and the value these could offer to alternate high performance professions.

Like all occupations, an esports career depends on financial and job security. Although some professional athletes in tier-1 (the highest level of competition) leagues within popular esports enjoy financial stability from yearly contracts (Esports Mention, 2019), athletes in less popular esports and aspiring gamers not yet competing in tier-1 leagues are not afforded this luxury. Additionally, outside tier-1 tournaments, prize money distribution is such that winners are more greatly rewarded at the expense of other participants (Coates and Parshakov, 2016). Moreover, protections are limited for esport athletes as they have not yet been able to unionize. This makes job security remarkably fragile, particularly given the high athlete replaceability, with extreme cases of top esport athletes being dropped from teams at post-victory celebrations (Van Allen, 2018).

To further compound these challenges, the average career of typical esports athletes' is remarkably short; with about one-in-five professional esport athletes' careers lasting 2 years or longer (Ward and Harmon, 2019). This short career length is largely due to the difficulty of becoming and remaining a top esport athlete, particularly given the volatility of team rosters. Esports performance is reliant on the ability to rapidly and accurately respond to complex visual stimuli, which begins to decline past 24 years of age (Thompson et al., 2014). As such, one's timeframe for peak esports performance is limited. The time commitment and rigor required for elite esports performance has resulted in many cases of burnout and injury, causing early retirement (Salo, 2017). Additionally, adolescent esport athletes often sacrifice educational opportunities to pursue their careers (Hollist, 2015), hampering their ability to pursue alternate careers post-retirement. In summary, there appears to be a narrow timeframe for financial success for esports athletes, who may jeopardize their post-retirement opportunities to take that window.

## Skills and Experience of an Esport Athlete

Despite the current pitfalls of an esports profession, esports athletes possess a unique range of specialized skills and experiences that we argue are highly sought after in many contemporary professions. Such attributes include digital intelligence, experience and expertise in prolonged human computer interaction performed in a seated posture, skillful and efficient communication, and perhaps most notably, enhanced cognitive abilities. In this section, we outline some of these traits that this population possess.

### Digital Intelligence and the Workplace Environment—Esport Athletes

Fundamentally, esports involve human-computer interactions with an adapting computer program to produce outcome-defining events within a virtual gameplay environment (Hamari and Sjöblom, 2017). Higher-level esport athletes perform faster and with more complexity than their less-skilled counterparts (Avontuur et al., 2013; Buckley et al., 2017). Esports are a type of high-performance computing that requires “digital intelligence” to provide a competitive advantage, such as knowledge of, and proficiency with, hardware components (Claypool and Claypool, 2007). Additionally, esports athletes undertake long continuous bouts (often >3 h) fixating on computer monitors in a seated posture during training and competition (DiFrancisco-Donoghue et al., 2019). It is well-established that this prolonged sitting can result in lower back discomfort and impaired vascular function (Dunk and Callaghan, 2010; Credeur et al., 2019). Moreover, frequent computer monitor use can lead to “computer vision syndrome,” associated with temporary eye discomfort (Blehm et al., 2005). Although little work has investigated these physiological effects in esports athletes, it may be that esports athletes have developed strategies to maintain performance despite these issues.

### Communication—Esport Athletes

Esport teams are a unique hybrid of a high-performance action team engaging in computed supported cooperative work (CSCW), a combination not regularly seen in more traditional team environments (Freeman and Wohn, 2019). Given that most esports are team-based, effective team cohesion and communication are essential for success. Communication within elite esports is overwhelmingly verbal (Lipovaya et al., 2018). To maximize efficiency and effectiveness of this communication, athletes must be proficient in utilizing rigid phraseologies (Lipovaya et al., 2018; Freeman and Wohn, 2019). Team strategies and individual roles must be effectively communicated prior-to and during competition to ensure successful performance (Lipovaya et al., 2018; Freeman and Wohn, 2019).

### Cognitive Skills—Esport Athletes

First Person Shooter (FPS) and Multiplayer Online Battle Arena (MOBA) games, which collectively comprise the majority of major esports and are known as action video-games (AVGs), are cognitively demanding (Campbell et al., 2018). The demand that esports places on athletes has resulted in a growing body of research demonstrating that gamers possess enhanced cognitive abilities compared to non-gamers (Kowal et al., 2018; Large et al., 2019); due to this, esport athletes have been referred to as “cognitive athletes” (Campbell et al., 2018).

Existing literature indicates that experienced AVG players (AVGPs) have enhanced spatial and temporal visual perception (Green and Bavelier, 2007; West et al., 2008; Li et al., 2009, 2010; Appelbaum et al., 2013). Further, AVGPs possess greater attentional resources, facilitating performance improvements on tasks with large attentional-demands (Bavelier et al., 2012a; Krishnan et al., 2013). Additionally, AVGPs can also better control their attentional resources (Chisholm et al., 2010; Mishra et al., 2011; Bavelier et al., 2012a; Chisholm and Kingstone, 2012; Green et al., 2012; Krishnan et al., 2013; Cain et al., 2014; Föcker et al., 2018) and allocate them over a wider visual field-of-view (Dale et al., 2019). Lastly, AVGPs demonstrate high capacity to integrate visual and auditory information (Donohue et al., 2010).

In addition to enhanced perception and attentional capabilities, AVGPs have been demonstrated to have faster overall response times across a diverse range of tasks, which is believed to represent general enhancements in cognitive throughput (Castel et al., 2005; West et al., 2008; Dye et al., 2009; Hubert-Wallander et al., 2011; Bavelier et al., 2012a; Green et al., 2012; Wu and Spence, 2013; Föcker et al., 2018). It has been suggested that these improved perceptual, attentional, and processing speed abilities provide gamers an enhanced capacity to learn as well (Bavelier et al., 2012b).

## The Translation of the Esport Skillset to Alternate Professions

Although their careers are tenuous and short-lived, the inherent skill-sets possessed by esports athletes are highly desirable in multiple contemporary professions. To demonstrate this, we queried the Occupational Information Network to determine professions sharing expertise and experience with esport athletes (O^*^net, queried 12/12/19). O^*^net is a free database of occupation-specific descriptors, developed with the sponsorship of the U.S. Department of Labor/Employment and Training Administration (USDOL/ETA), to help jobseekers match careers to their skill-sets. Within the database, almost 1,000 professions are ranked according to defined categories of abilities/experience; we queried those categories related to the expertise of esport athletes.

**Figure 1:**
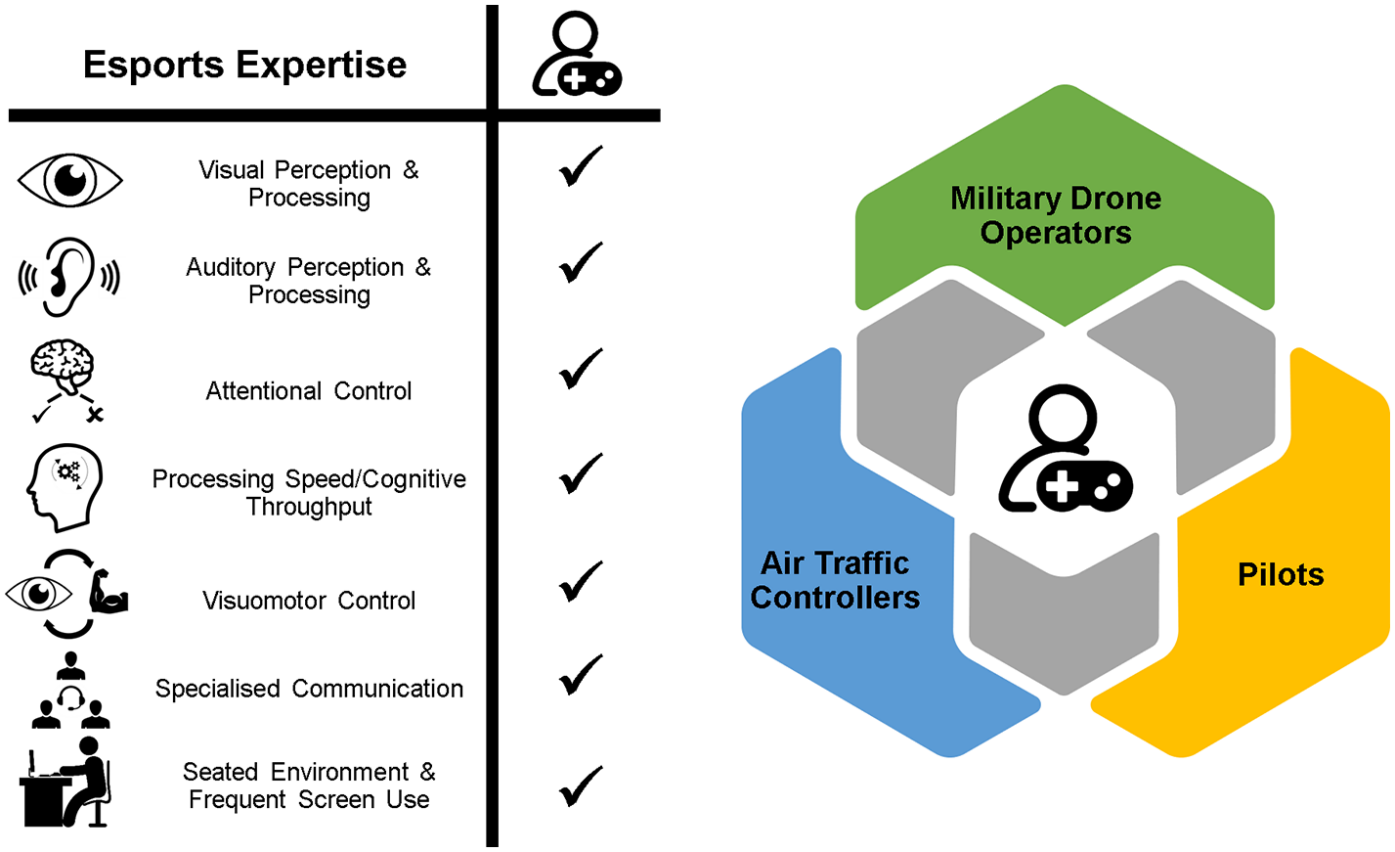
Prominent skills and experiences possessed by esport athletes (left) and a Venn diagram (right) visually demonstrating the mutual desire for esport-related skill and experience by military UAS (drone) operators, air traffic controllers, and pilots.

Following our searches, two professions very regularly (over 40%) appeared (top 30 most relevant occupations; see Supplementary Table 1 for search details): aircraft pilots (hereafter simply referred to as “pilots”) and air traffic controllers (ATCs). Additionally, previous research demonstrating comparable performance of AVGPs to military combat pilots on simulated Unmanned Aerial System (UAS) operations (McKinley et al., 2011) led us to include this profession. The following sections explore how the skill-sets and experience required for success in esports could be highly valued in these professions (see Figure 1).

### Digital Intelligence and Workplace Environment—Pilots, ATCs and UAS Operators

Expert interfacing with complex computerized systems is essential for the outlined professions, with ATCs and UAS operators, in a similar manner to esports, using computer monitors as primary outputs. For ATCs, the quality of such human-computer interface interactions is integral to overall performance (Chang and Yeh, 2010). Furthermore, McKinley et al. (2011) noted that gamers were highly proficient at using “game-like” UAS interfaces, supporting the performance benefits of interface familiarity. All of the outlined professions are also performed while seated, with pilots specifically remaining in such a posture for several hours at a time (Lusted et al., 1994). While pilots do not fixate on screens for prolonged periods, ATCs and UAS operators do, and may experience “computer vision syndrome” as a result. Given that esport athletes often perform during long bouts of sitting and screen fixation, they may be better suited to maintaining high task performance in jobs that appear to also have these challenges.

### Communication—Pilots, ATCs and UAS Operators

Pilots and ATCs must work synergistically to maximize safety in operations where communication, much like esports, is primarily digital (CSCW). Very specific phraseology is used in aviation to optimize communication efficiency (Campbell-Laird, 2004). Such consistency is vital, as coordination and communication errors are the leading cause of air traffic accidents (Isaac and Ruitenberg, 2017). Similarly, UAS operators are required to have frequent and concise verbal communication with ATCs, ground units, and other aircraft (McKinley et al., 2011).

### Cognitive Skills—Pilots, ATCs and UAS Operators

The key similarities between esports athletes, pilots, ATCs and UAS operators lie in their cognitive abilities. Rapid identification (perception and attention) and processing (cognitive throughput) of information is vital for safety and operational performance. For ATCs and pilots, timely recognition and response to warnings on one of many displays (often in visual peripheries) can prevent hundreds of casualties, while timely localization and action toward a target can define operational success or failure among UAS operators. Important information for all three professions is invariably concealed among an array or “clutter” of stimuli, rendering attentional control as critical. Moreover, individuals in these professions are often required to simultaneously attend to multiple stimuli at once (multitasking); particularly ATCs, who constantly must manage airspaces containing numerous aircraft. Information may be visual (displays), haptic and auditory (alarms, warnings, and verbal), placing importance on multisensory integration.

Given such intense demands, attentional errors are among the most common errors for ATCs (Pape et al., 2001), and cognitive processing/decision making errors constitute most “pilot error” accidents (Adams and Ericsson, 2000); both of which can result in numerous casualties. To mitigate such risks, pre-training assessments for these professions regularly include assessing these aforementioned cognitive attributes; given the cognitive proficiency of esport athletes, they may perform well on such tests, demonstrating high suitability for these professions.

It must be acknowledged that the skill-sets/experiences possessed by esport athletes are not exclusively beneficial for pilots, ATCs, and UAS operators, and may be favorable for any occupations which share similar workplace demands, communication, and cognitive requirements.

## Field Challenge

The rise of esports has resulted in the emergence of a population of uniquely skilled young individuals. These “cognitive athletes” can quickly perceive and process large amounts of information, while simultaneously demonstrating better attentional control. Moreover, they are strong communicators and work well in team environments, particularly through digital means and in high-performance computing contexts. Lastly, they are notably proficient with human-computer interfaces, and are experienced with working in a seated posture for extended periods. Unfortunately, given the nature of esports as a profession, most esport athletes experience a short, financially unstable career, with limited post-retirement opportunities.

Here, we have highlighted the shared importance of the unique skills and experiences possessed by esport athletes and how they may be preferentially valued for three exemplar professions; pilots, ATCs, and military UAS operators. High-performance in these three professions is critical, as errors can pose large financial and human costs. Overall, this work poses a challenge to the esports, scientific and industrial communities, to demonstrate how best to leverage the unique abilities of esports athletes to facilitate their life after esports and add value to professions seeking individuals with these unique skillsets. Doing so could result in more suitable personnel occupying the abovementioned industries and would be highly beneficial to the distinctly vulnerable population of esport athletes.

## Author Contributions

All authors contributed to the conception of the field challenge. TS wrote the manuscript. All authors contributed to manuscript revision, read, and approved the submitted version.

## Conflict of Interest

The authors declare that the research was conducted in the absence of any commercial or financial relationships that could be construed as a potential conflict of interest.
